# Systemic lupus erythematosus-related infections in pregnancy: a cross-sectional bibliometric analysis

**DOI:** 10.1007/s00296-026-06086-4

**Published:** 2026-03-23

**Authors:** Dinara Yerlanova, Maidan Mukhamediyarov, Olena Zimba, Mariusz Korkosz, Burhan Fatih Kocyigit

**Affiliations:** 1 Department of Social Health Insurance and Public Health, South Kazakhstan Medical Academy, Shymkent, Kazakhstan; 2 Department of Chemical Disciplines, Biology and Biochemistry, South Kazakhstan Medical Academy, Shymkent, Kazakhstan; 3 Department of Rheumatology, Immunology and Internal Medicine, University Hospital in Kraków, Kraków, Poland; 4 National Institute of Geriatrics, Rheumatology and Rehabilitation, Warsaw, Poland; 5 Department of Internal Medicine N2, Danylo Halytsky Lviv National Medical University, Lviv, Ukraine; 6 Department of Rheumatology and Immunology, Jagiellonian University Medical College, Jakubowskiego 2 Str., 30-688, Kraków, Poland; 7 Department of Physical Medicine and Rehabilitation, University of Health Sciences, Adana Health Practice and Research Center, Adana, Türkiye

**Keywords:** Systemic lupus erythematosus, Pregnancy, Infections, Bibliometrics, Bibliometric analyses, Pregnancy complications, Databases, Bibliographic

## Abstract

Pregnancy raises the risk of maternal and fetal complications in systemic lupus erythematosus (SLE) patients due to physiological and immunological changes, with infections standing out as a significant concern. It is important to conduct a comprehensive examination of the structure and trends in the scientific literature of this field. This bibliometric study analyzed publications on SLE-related infections during pregnancy using the Scopus database. The search was conducted on January 15, 2026, using the keywords “Systemic Lupus Erythematosus”, “Pregnancy”, and “Infection” in the title, abstract, and keyword fields. The analysis included an examination of publication distribution and trends over time using linear regression. Data on countries, authors, institutions, funding sources, journals, document types, and keywords of the articles were collected. A total of 994 publications were included in the analysis. The annual number of publications increased significantly over the years and the publication output peaked in 2024 (*n* = 73) (R² = 0.664, *p* < 0.001). In total, 71 countries contributed to the literature, with 28 classified as main active countries (≥ 1% of total output). The majority of document types were articles (*n* = 528) and reviews (*n* = 354). In terms of publication volume, the United States (*n* = 299), the United Kingdom (*n* = 101), and Italy (*n* = 83) were prominent, whereas in terms of citations per paper, Hungary (170.4), Greece (137.23), and the Netherlands (116.55) exhibited the highest impact. The most productive journals included *Lupus* (*n* = 36), *Frontiers in Immunology* (*n* = 20), and *Annals of the Rheumatic Diseases* (*n* = 14). This analysis reveals a significant upward trend in the scientific literature in recent years and the increasing clinical importance of the subject.

## Introduction

Systemic lupus erythematosus (SLE) is a diverse, complex, and multisystemic autoimmune disorder predominantly impacting women of reproductive age [1]. SLE impacts the pregnancy period, increasing the risk of maternal and fetal complications [2]. Patients with SLE exhibit an elevated susceptibility to infections compared with the general population, attributable to compromised immune function, organ involvement, and regular use of immunosuppressive therapies [3]. The heightened risk of infection is particularly evident during pregnancy, a phase characterized by substantial physiological and immunological alterations. The immune adaptation mechanisms that emerge during pregnancy, along with the complex characteristics of SLE, may substantially increase the risk of infections during pregnancy [4, 5].

Bibliometric analysis is an approach that aims to assess the framework, developmental course, and research trends within a particular scientific domain using quantitative data [6, 7]. Analyzing publication statistics, citation metrics, contributions by countries, institutions, and authors, and keyword distributions facilitates understanding of scientific output, impact levels, and research trends within a topic [8, 9]. This approach promotes systematic mapping of the knowledge in the literature, identification of prominent research institutions and collaborations, and forecasting of prospective research domains. In recent years, bibliometric investigations, particularly in medicine and the health sciences, have been widely used to assess research dynamics [10, 11].

This study aimed to conduct a thorough bibliometric analysis of the scientific literature on SLE-related infections during pregnancy. The analysis encompassed the annual distribution of publications, temporal trends, contributions by countries, institutions, and authors, the most prolific journals, funding sources, and keyword patterns. The objective was to ensure that the findings enhance comprehension of research trends within this domain and provide directions for subsequent studies.

## Materials and methods

### Search strategy

This study utilized the Scopus database to examine scientific publications on infections during pregnancy in SLE. Publication data were acquired from a search conducted on January 15, 2026. The search strategy was established employing the keywords “Systemic Lupus Erythematosus” AND “Pregnancy” AND “Infection,” which were queried in the title, abstract, and/or keyword fields (TITLE–ABSTRACT–KEYWORDS). No time frame constrained the study; therefore, all studies published from the earliest date in 1964 through to 2026 were incorporated into the analysis. Furthermore, all publication types indexed by the Scopus database, including articles, reviews, conference papers, and other document types, were assessed [12, 13]. This search approach was implemented to ensure a thorough representation of the literature on the topic, minimize the risk of omitting relevant publications, and comprehensively assess the historical development. No language restrictions were set, thereby guaranteeing the incorporation of works published in different languages in the analysis. This approach sought to offer a more comprehensive perspective on the subject and to assess publications on the topic thoroughly.

### Data collection

All publications retrieved from the Scopus database in accordance with the specified search criteria were recorded for analysis. Within the scope of the investigation, the annual distribution of publications from 1964 to 2026 was documented. By assessing the distribution of publications across countries, the total number of contributing countries was determined. Countries that accounted for 1% or more of total publications were designated as “main active countries” [14, 15]. For these countries, total number of publications, total number of citations, and average citations per paper (citations per publication) were calculated and recorded. The ten leading journals in this field were identified. Total citations and average citations per publication for each journal were noted. The CiteScore 2024 data for the ten leading journals was obtained from the Scopus database. CiteScore is a metric that quantifies the average citations received by papers published in a journal [16]. Distribution of publications by document type was examined, and number of publications for each category was documented. The ten most prolific institutions contributing to the literature were picked. The ten most frequently used keywords were identified and listed by frequency. The five most prolific authors in literature were identified, and their publication counts were documented.

### Statistical analyses

Data are expressed as numbers (n). Graphs and other visual representations were generated using Microsoft Excel. The trend in the number of publications over the years was analyzed using linear regression in the Statistical Package for the Social Sciences (SPSS) version 20.0 (SPSS Inc., Chicago, IL, USA). A p-value less than 0.05 was considered statistically significant.

## Results

**Fig. 1:**
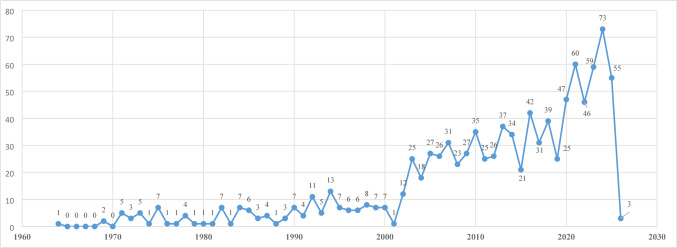
Distribution of the number of publications over the years

In accordance with the search strategy outlined in the Methods section, a total of 994 documents were incorporated into the bibliometric analysis. The initial publication in the field was released in 1964, and data from 1964 to 2026 were examined to assess publication trends over time. When analyzing the distribution of annual publication counts over time, it was observed that publication output peaked in 2024 (*n* = 73). Linear regression analysis (trend analysis) conducted to assess the change in the annual number of publications from 1964 to 2026 demonstrated that the model was statistically significant (R² = 0.664, *p* < 0.001), with the year variable showing a positive association with publication output (B = 0.80, 95% CI 0.66–0.94). Analysis of the regression coefficients indicated that the year variable exerted a positive, statistically significant effect on the number of publications (B = 0.80, t = 10.98, *p* < 0.001). These findings demonstrate that the number of publications increased by approximately 0.8 units per year throughout the study period, revealing a statistically significant upward trend in this field (Fig. 1).

**Fig. 2:**
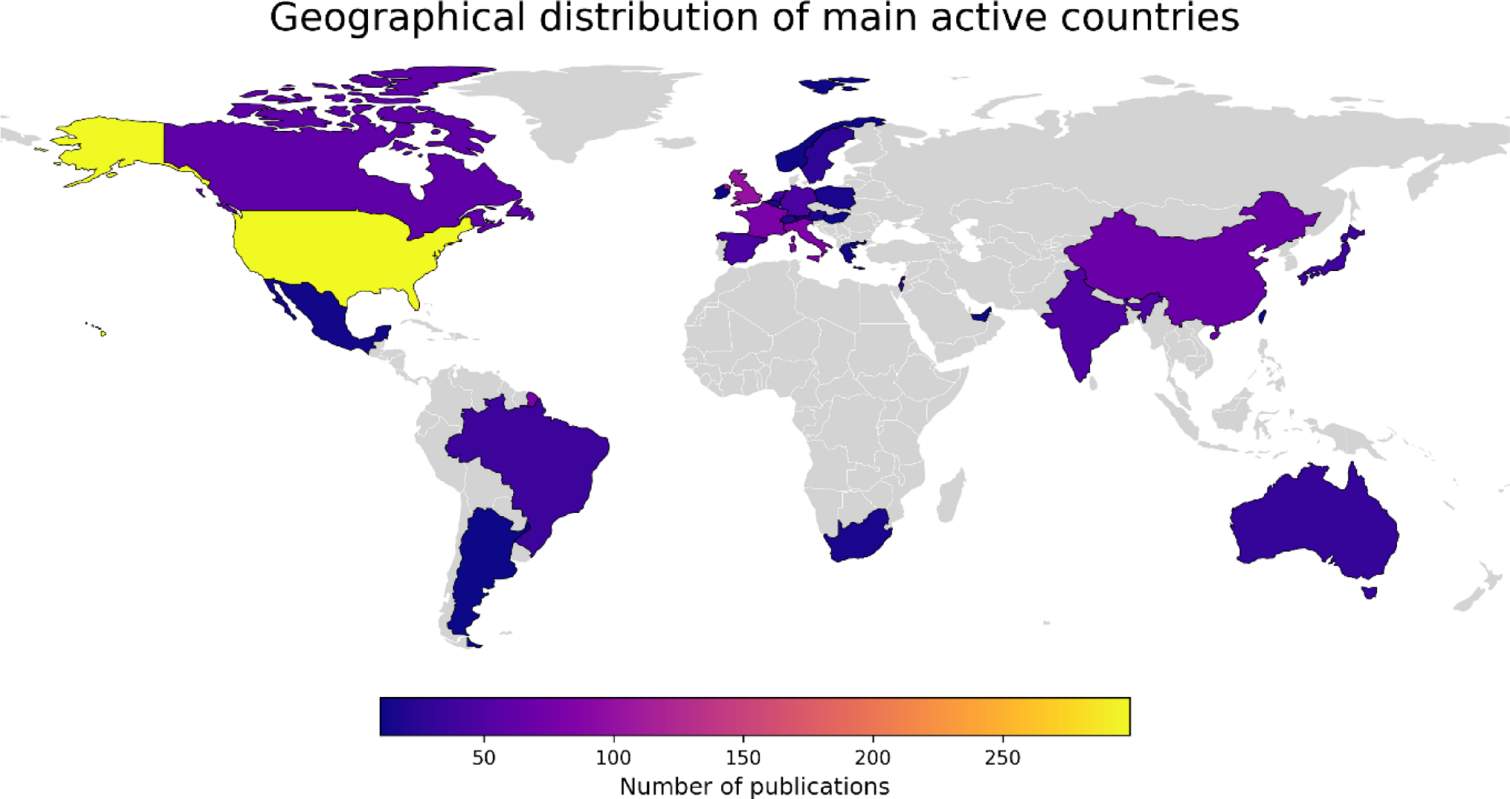
Geographical distribution of main active countries by number of publications

When the distribution of publications by countries was assessed, 71 countries were identified as contributing to the literature. Countries providing 1% or more of the total publications were assigned as “main active countries”, with 28 countries identified within this category (Table 1) (Fig. 2). Based on the number of papers, the leading three countries were the United States (*n* = 299), the United Kingdom (*n* = 101), and Italy (*n* = 83). When assessed by citations per publication, the top three were Hungary (170.4), Greece (137.23), and the Netherlands (116.55).

**Table 1:** Publication output and citation impact of the main active countries

Country	Number of Publications	Total Citations	Citations per Publication
United States	299	19,338	64.68
United Kingdom	101	8569	84.84
Italy	83	6295	75.84
France	78	5549	71.14
China	67	1475	22.01
Canada	59	3094	52.44
India	50	904	18.08
Spain	45	2400	53.33
Germany	45	3389	75.31
Netherlands	40	4662	116.55
Japan	38	555	14.61
Brazil	35	1086	31.03
Australia	31	762	24.58
Israel	30	2918	97.27
Sweden	28	1914	68.36
Switzerland	21	1079	51.38
Austria	18	1767	98.17
Belgium	16	1698	106.13
South Africa	15	145	9.67
Poland	15	371	24.73
Mexico	13	432	33.23
Greece	13	1784	137.23
Norway	12	415	34.58
Argentina	12	578	48.17
United Arab Emirates	11	231	21.00
Taiwan	11	92	8.36
Hungary	10	1704	170.40
Ireland	10	912	91.20

Countries that contributed ≥ 1% of total publications were classified as main active countries. Citation impact is expressed as average citations per publication

The top ten journals producing the most publications in this field are the following: *Lupus* (*n* = 36), *Frontiers in Immunology* (*n* = 20), *Annals of the Rheumatic Diseases* (*n* = 14), *Autoimmunity Reviews* (*n* = 12), *Seminars in Arthritis and Rheumatism* (*n* = 10), *Journal of Rheumatology* (*n* = 10), *American Journal of Obstetrics and Gynecology* (*n* = 9), *Medicine (United States)* (*n* = 8), *Clinical Rheumatology* (*n* = 8), and *Blood* (*n* = 8) (Table 2). Based on citations per publication, the top three journals are *Annals of the Rheumatic Diseases* (222.21), *Blood* (173.5), and *American Journal of Obstetrics and Gynecology* (155.22). According to CiteScore 2024 rankings, the top three journals are *Annals of the Rheumatic Diseases* (33.2), *Blood* (23), and *Autoimmunity Reviews* (16.8).

**Table 2:** Top ten sources by number of publications

Source	Number of Publications	Citations per Publication	CiteScore 2024
*Lupus*	36	26.25	4.1
*Frontiers in Immunology*	20	50.75	10.8
*Annals of the Rheumatic Diseases*	14	222.21	33.2
*Autoimmunity Reviews*	12	52.25	16.8
*Seminars in Arthritis and Rheumatism*	10	83	8.1
*Journal of Rheumatology*	10	32.1	5.9
*American Journal of Obstetrics and Gynecology*	9	155.22	14.2
*Medicine (United States)*	8	22	2.5
*Clinical Rheumatology*	8	7.38	5.9
*Blood*	8	173.5	23

Journals are ranked by publication volume. Citation impact is presented as average citations per publication, and CiteScore 2024 values are provided to reflect journal-level scientific influence

**Fig. 3:**
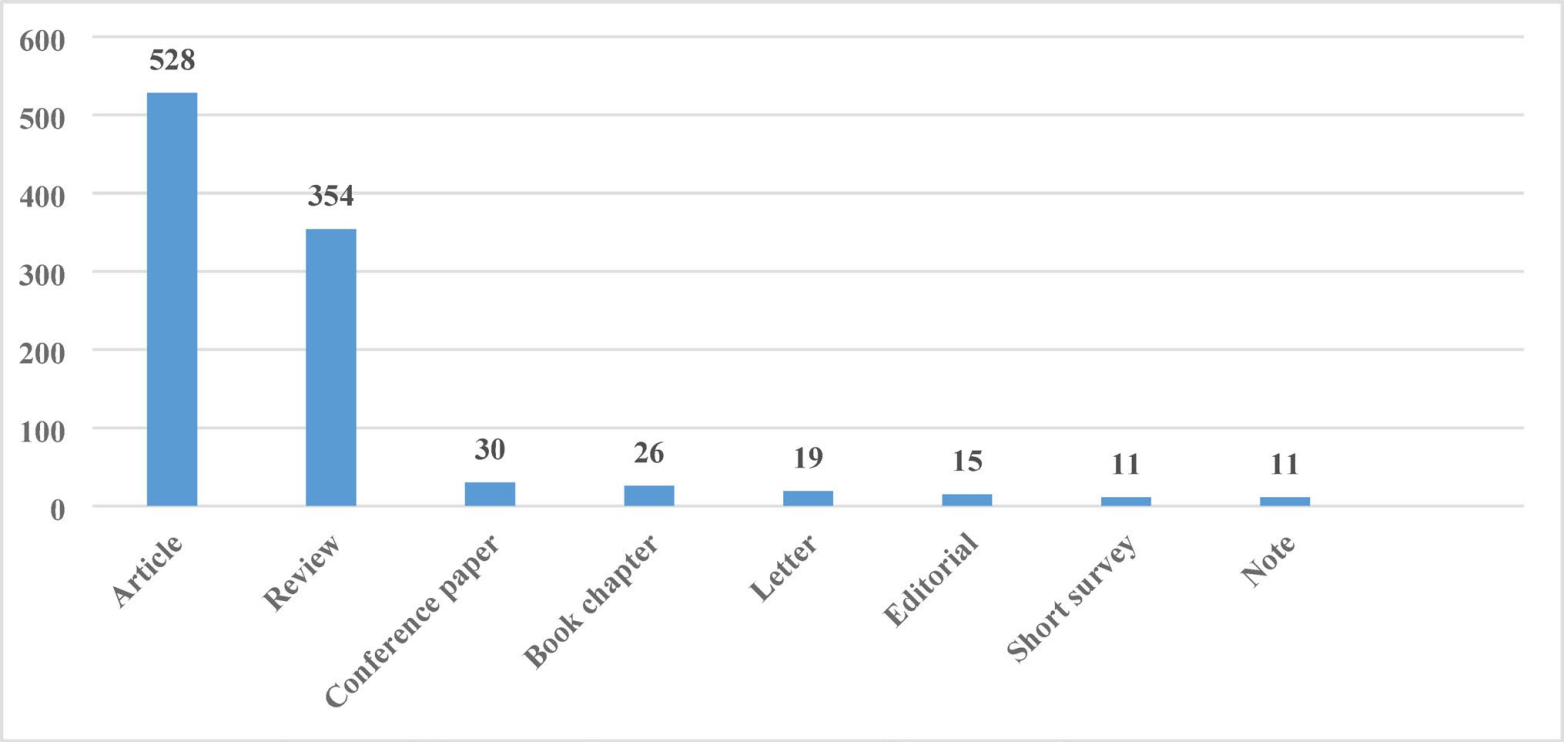
Distribution of publication numbers according to document type

Analysis by document type revealed that articles (*n* = 528) constituted the vast majority of publications, followed by reviews (*n* = 354). Conference papers (*n* = 30), book chapters (*n* = 26), letters (*n* = 19), editorials (*n* = 15), short surveys (*n* = 11), and notes (*n* = 11) had a limited share in the literature (Fig. 3).

Among the most prolific authors, Shoenfeld, Y. (*n* = 13) ranked first, followed by Tincani, (A) (*n* = 10). Cervera, R. (*n* = 8), Godeau, P. (*n* = 8), and Wechsler, (B) (*n* = 8) were identified as other leading prolific authors in this field (Table 3).

**Table 3:** Five most prolific authors and number of publications

Author	Number of Publications
Shoenfeld, Y.	13
Tincani, A.	10
Cervera, R.	8
Godeau, P.	8
Wechsler, B.	8

Keyword analysis revealed that the ten most frequently used keywords were Human (*n* = 937), Systemic Lupus Erythematosus (*n* = 880), Pregnancy (*n* = 761), Female (*n* = 643), Humans (n ​​= 609), Article (*n* = 476), Adult (*n* = 410), Review (*n* = 371), Priority Journal (*n* = 331), and Infection (*n* = 280) (Table 4).

**Table 4:** Top ten keywords by number of occurrences

Keyword	Number of Occurrences
Human	937
Systemic Lupus Erythematosus	880
Pregnancy	761
Female	643
Humans	609
Article	476
Adult	410
Review	371
Priority Journal	331
Infection	280

When the distribution of publications by institution was examined, the University of Toronto (*n* = 22) stood out as the institution with the most publications, followed by Università degli Studi di Milano (*n* = 19) and Harvard Medical School (*n* = 18). Other leading institutions included Hôpital Universitaire Pitié Salpêtrière (*n* = 17), University College London (*n* = 17), Hospital for Special Surgery – New York (*n* = 17), Inserm (*n* = 16), Tel Aviv University (*n* = 16), Weill Cornell Medicine (*n* = 15), and Hospital Clínic de Barcelona (*n* = 15) (Table 5).

**Table 5:** Top ten institutions by number of publications

Institution	Number of Publications	Country	
University of Toronto	22	Canada	
Università degli Studi di Milano	19	Italy	
Harvard Medical School	18	United States	
Hôpital Universitaire Pitié Salpêtrière	17	France	
University College London	17	United Kingdom	
Hospital for Special Surgery – New York	17	United States	
Inserm	16	France	
Tel Aviv University	16	Israel	
Weill Cornell Medicine	15	United States	
Hospital Clínic de Barcelona	15	Spain	

When funding sources were examined, the National Institutes of Health (NIH, United States; *n* = 25) stood out as the organization supporting the most publications. This was followed by the National Natural Science Foundation of China (NSFC, China; *n* = 19) and the National Institute of Arthritis and Musculoskeletal and Skin Diseases (NIAMS, United States; *n* = 18). Other prominent funders included the Japan Society for the Promotion of Science (JSPS, Japan; *n* = 11) and the Medical Research Council (MRC, United Kingdom; *n* = 9) (Table 6).

**Table 6:** Top five funding sources

Funding Sources	Country	Number of Supported Publications
National Institutes of Health (NIH)	United States	25
National Natural Science Foundation of China (NSFC)	China	19
National Institute of Arthritis and Musculoskeletal and Skin Diseases (NIAMS)	United States	18
Japan Society for the Promotion of Science (JSPS)	Japan	11
Medical Research Council (MRC)	United Kingdom	9

## Discussion

This bibliometric analysis revealed a significant upward trend in the scientific literature on SLE-related infections during pregnancy, underscoring the expanding clinical and scientific importance of this field. While the United States, the United Kingdom, and Italy led in total number of papers, Hungary, Greece, and the Netherlands showed high levels of scientific impact citation-wise. Furthermore, analyses of journals, institutions, authors, and funding sources revealed that research in this field is centered on particular centers and collaborative efforts, while the distribution of keywords confirmed that themes related to pregnancy, infection, and women’s health predominate in the literature.

Recent clinical data underscore the importance of improved pregnancy care in SLE. A nationwide Finnish case-control study indicated generally moderate pregnancy outcomes, although it revealed elevated rates of urgent cesarean deliveries and neonatal critical care unit admissions among women with SLE compared to controls [17]. Moreover, evidence from specialized pregnancy and rheumatic disease clinics suggests that organized pre-pregnancy planning may mitigate disease flares and medication changes during pregnancy [18], likely contributing to the increasing research interest identified in this bibliometric analysis.

The rising trend in publication volume reflects the growing clinical and scientific relevance of SLE-related infections during pregnancy. The notable rise in publication output, particularly in recent years, may be attributed to the heightened emphasis on pregnancy counseling and the necessity for a deeper comprehension of the infection risks faced by this patient group [19, 20]. Furthermore, the increased use of immunosuppressive and biologic agents in SLE treatment may have contributed to the infection risk during pregnancy, becoming a more complex clinical issue [21, 22]. This situation may have heightened the need for evidence-based strategies to prevent, diagnose, and manage infections, possibly encouraging research in this area.

The extensive geographical dispersion of papers, with contributions from 71 countries, underscores the global relevance of SLE-related infections during pregnancy. However, the identification of 28 main active countries reveals that scientific activity is concentrated in certain regions. The prominence of the United States, the United Kingdom, and Italy in publication output certainly indicates robust research systems, specialized referral institutions, and sustained funding [23]. Hungary, Greece, and the Netherlands exhibited the most citations per paper. The elevated citation rates in such countries may be ascribed to an emphasis on hypothesis-centered investigations, pivotal clinical trials, or research that feeds guidelines and significantly impacts future studies.

High number of papers in *Lupus*, *Frontiers in Immunology*, and *Annals of the Rheumatic Diseases* suggests that this issue is predominantly addressed within the fields of rheumatology and immunology. The fact that *Annals of the Rheumatic Diseases*, *Blood*, and *the American Journal of Obstetrics and Gynecology* rank high in the evaluation of citations per publication suggests that studies in these journals have substantial scientific influence on the literature. The inclusion of *Blood* and *the American Journal of Obstetrics and Gynecology*, in particular, suggests that infections related to SLE during pregnancy are not restricted to the field of rheumatology.

Keyword analysis indicates that the literature was predominantly organized around clinical research on human subjects, female characteristics, and pregnancy. The high frequency of the terms “Systemic Lupus Erythematosus”, “Pregnancy”, and “Infection” indicates that the study’s topic was clearly and consistently handled in the literature.

The institutional distribution shows that publications on SLE-related infections in pregnancy were concentrated in institutions with strong academic infrastructure and multidisciplinary approaches. The prominence of institutions such as the University of Toronto, Università degli Studi di Milano, and Harvard Medical School suggests that these centers have strong research traditions in rheumatology, obstetrics, and immunology.

An analysis of funding sources suggests that most research is conducted with significant public support at the national level. The prominence of funding institutions, notably those in the United States (NIH and NIAMS), reflects the presence of long-term research plans in this subject [24, 25]. Contributions from national research funds in China, Japan, and the United Kingdom suggest that this topic has emerged as a global research priority.

### Limitations

The study has several limitations. First, the analysis was restricted to the Scopus database. Therefore, certain papers indexed by other reputable databases may have been excluded from the research. However, given the broad scope of the Scopus database, the current data correctly reflect research trends in the field [26]. Second, bibliometric analyses rely on quantitative indicators, and the scientific quality, methodological soundness, and clinical influence of papers are not examined. Citation counts and bibliometric indicators reflect the influence of publications, but they are dynamic metrics that vary over time. Although no language restrictions were set in the study, publications in languages other than English received fewer citations, which may have influenced the interpretation of specific results. Finally, this study has a cross-sectional design, with analyses based on data obtained on a particular date. As a result, future publications or changes in citation numbers over time are not captured in the current analysis.

From an organizational standpoint, this bibliometric analysis may inform the design of rheumatological and obstetric care for pregnant women with SLE, underscoring the importance of managing infection risk during pregnancy. The presence of numerous high-impact studies in multidisciplinary centers underscores the need for coordinated care approaches that integrate expertise in rheumatology, obstetrics, and infectology, along with structured pre-pregnancy counseling, to improve outcomes.

## Conclusion

This bibliometric analysis demonstrated an upward trend in the scientific literature on SLE-related infections in pregnancy over time, reflecting the issue’s growing clinical and scientific significance. The outcomes indicated that research output is predominantly concentrated in certain countries and institutions. Analysis of journals, authors, institutions, and funding sources revealed that SLE-related infections during pregnancy have emerged as a multidisciplinary research domain encompassing rheumatology, immunology, and obstetrics. It is anticipated that the findings will inform future clinical and epidemiological investigations, support the identification of research gaps, and facilitate the development of strategies for more effective management of infection risk within this patient group.

## Data Availability

Raw data can be provided by the corresponding author upon reasonable request.

## Declarations

### Responsibility for the integrity and accuracy

All authors take full responsibility for the integrity and accuracy of all aspects of the work.
